# Crackle template based metallic mesh with highly homogeneous light transmission for high-performance transparent EMI shielding

**DOI:** 10.1038/srep25601

**Published:** 2016-05-06

**Authors:** Yu Han, Jie Lin, Yuxuan Liu, Hao Fu, Yuan Ma, Peng Jin, Jiubin Tan

**Affiliations:** 1 Center of Ultra-precision Optoelectronic Instrument, Harbin Institute of Technology, Harbin 150080, P. R. China; 2 School of Energy Science and Engineering, Harbin Institute of Technology, Harbin 150001, P. R. China; 3 Department of Electrical and Computer Engineering, Dalhousie University, Halifax, NS B3H 4R2, Canada

## Abstract

Our daily electromagnetic environment is becoming increasingly complex with the rapid development of consumer electronics and wireless communication technologies, which in turn necessitates the development of electromagnetic interference (EMI) shielding, especially for transparent components. We engineered a transparent EMI shielding film with crack-template based metallic mesh (CT-MM) that shows highly homogeneous light transmission and strong microwave shielding efficacy. The CT-MM film is fabricated using a cost-effective lift-off method based on a crackle template. It achieves a shielding effectiveness of ~26 dB, optical transmittance of ~91% and negligible impact on optical imaging performance. Moreover, high–quality CT-MM film is demonstrated on a large–calibre spherical surface. These excellent properties of CT-MM film, together with its advantages of facile large-area fabrication and scalability in processing on multi-shaped substrates, make CT-MM a powerful technology for transparent EMI shielding in practical applications.

Electromagnetic interference (EMI) generally consists of many unwanted radiative signals that could introduce noise or malfunction to electronic devices or even cause damage to the human body. The rapid growth of the electronic device market not only overcrowds the spectral bands to the different communication channels but also increases the density of electromagnetic emitters in the environment. Thus, many electronic facilities must be equipped with high-efficiency EMI shielding for microwave range interference from the ambient environment^1^,^2^,^3^,^4^,^5^. The realization of EMI shielding in the optoelectronic equipment, especially for optical windows or domes, is still a great challenge. High optical transmittance and strong EMI shielding effectiveness (SE) are the two basic requirements that drive the development of transparent EMI shielding materials for practical applications^6^,^7^,^8^,^9^. More importantly, under harsh environments, transparent EMI shielding materials with a broadband transmission spectrum (Vis-IR) and stable imaging quality are attractive for a wide range of civil, commercial, military, and aerospace applications (e.g., electro-optical sensor pods)^10^,^11^. Practical applications also call for good scalability and low cost fabrication techniques. The Development of a high–performance EMI material with all its desirable features remains a great technological challenge.

To date, indium tin oxide (ITO), the best transparent conductive oxide (TCO)^12^,^13^, shows good visible transparency and modest EM wave attenuation performance^14^. However, the limited abundance of indium, along with the stiffness and brittleness properties of ITO, are barriers to the progress of ITO^15^,^16^. In addition, TCOs are poor UV and IR transmitters, leading to a band-limited transmission spectrum^17^. Carbon-based materials, including carbon nanotubes (CNTs) and graphene, show superiority in terms of their flexibility and high electrical conductivity^18^,^19^,^20^. They can be used to achieve high transmittance and strong EMI SE^9^,^10^,^21^. However, the fabrication of highly homogeneous CNT or graphene films with excellent optical and electrical properties, such as transmittance higher than 90% and sheet resistance within 10 Ω/sq, is limited to small-scale and planar surfaces^22^,^23^,^24^,^25^. In summary, all these materials suffer from a severe reciprocal restraint between optical transmissivity and electrical conductivity.

Metallic networks, such as metallic meshes and metallic nanowires (MNWs), can alleviate the restraint between transmissivity and conductivity to some extent and even widen the optical transmission spectrum because of the hollow metal structures^8^,^26^. The optical transmittance of metallic networks shows material independence in the full-wave band. The key merits of MNW films are simple solution processing, large area amenability and stability towards flexing. However, the stacked MNWs have intrinsic drawbacks of percolation and large contact resistances between wires, exactly like CNTs^27^,^28^,^29^. Moreover, the densely distributed nanowires result in compromised transmission of UV/IR light^8^. In contrast, regular metallic meshes can perfectly eliminate the percolation between wires, contact resistances at grid junctions and dense distribution of wires, attributed to the controllable design and patterning of metal wires. Conductive metallic meshes with submillimetre grid spacing and microscale line width exhibit free transmission of the Vis-IR spectrum and strong reflection of microwaves simultaneously^30^. Although periodic metallic mesh films can indeed realize highly broadband light transmission and strong EMI SE, stray light interference caused by diffraction superposition of periodic structures would significantly influence the imaging quality through the film^11^,^31^. Additionally, metallic meshes fabricated by photolithography are of limited linewidth and scale^32^,^33^. Extra polymer residual layers (poor UV/IR transmission) will be introduced by a nanoimprint lithography process^34^,^35^. The fabrication of large-area and nonplanar metallic mesh films is still costly and time consuming. Therefore, conventional photolithographically fabricated metallic mesh film is not the solution for high–performance EMI shielding films.

In the following, we report a technology for preparing large–area metallic mesh films by a facile lift-off method based on a drying-mediated self-forming template and their application in transparent EMI shielding. A novel emulsion with controllable cracking features was developed for the preparation of large-scale crackle templates on substrates with different shapes. By simply depositing a conductive metal on the crackle templates and then removing the sacrificial templates, crackle template based metallic mesh (CT-MM) films were obtained. The investigation into the optical transmission and EMI shielding performance of the resulting CT-MM films was conducted. The final results showed that CT-MM is a promising candidate for high-performance EMI shielding materials with high transmittance in the UV-Vis-IR spectrum, strong EMI shielding efficacy, negligible influence on optical imaging, and outstanding scalabilities and adaptabilities for various shapes, suitable for modern optoelectronic systems.

## Results

### Fabrication of metallic mesh film based on crackle template

**Figure 1:**
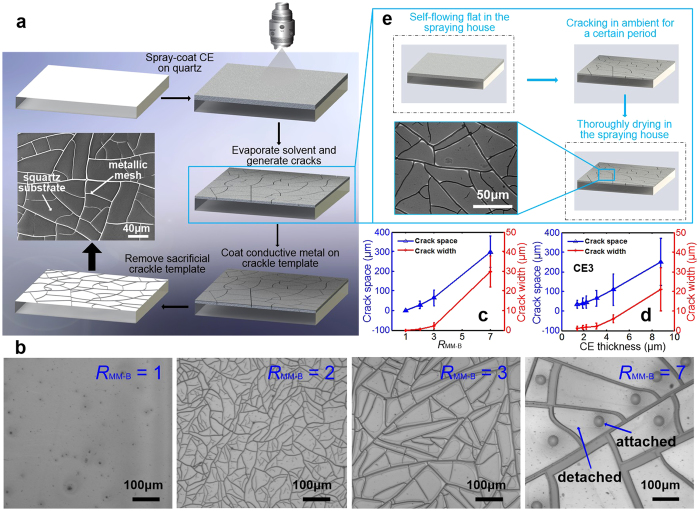
Fabrication process and optical and SEM characterizations of the CT-MM film. (**a**) Schematic illustration of the fabrication steps for a large-area CT-MM film. The SEM image shows the highly connected metallic networks. (**b**) Optical images of crackle templates with different values of *R*_MM-B_, showing that the crack width and cell size can be controlled by the amount of monomers. (**c**) Experimental curves of crack width and crack spacing of the dried CEs with different values of *R*_MM-B_. (**d**) Variations in crack width and crack spacing with respect to coating thickness. (**e**) Drying process of crackle template. Inset: SEM image showing the fine cracks with ignorable buckling.

The fabrication process of the CT-MM film is illustrated in Fig. 1a, which is a low-cost method based on a self-forming crackle template and lift-off technique^36^,^37^. The method includes mask layer coating, crackle template formation, conductive layer (silver) deposition and removal of the sacrificial layer. We introduced an adjustable emulsion, a large-area coating strategy and a drying-mediated method into the controllable fabrication of the crackle template. A CE was first developed using conventional monomer polymerization (detailed in Methods and Supplementary Note 1)^38^. In the CE preparation, the match between the hard monomer (methylmethacrylate, MMA) and soft monomer (butyl acrylate, BA) dominates the cracking property of CE. Considering the mass ratio *R*_MM-B_ between MMA and BA, CEs with different values of *R*_MM-B_ (i.e., 1, 2, 3 and 7) but the same total amount of monomers were prepared. They are termed CE1, CE2, CE3 and CE7, respectively. The optical images in Fig. 1b show a series of crackle templates obtained with the above mentioned CEs. The CE with low *R*_MM-B_ =  1 did not crack at all but formed a good CE film after drying. Such a recipe could be useful for paint and ink products^39^,^40^. With the increase in *R*_MM-B_, MMA becomes dominant in the CE solution. When *R*_MM-B_ reaches 2, the dried CE film shows disordered and poorly connected cracks on the whole surface. There are abundant disconnected residual cracks within crack cells. However, submillimetre and severely delaminated cracks can be observed if an unreasonably high amount of MMA, such as 30%, is added. For crackle template formed by CE7, only small, black pads remained in contact between the cracked CE cells and the substrate. The hardness of dried CE films varies with the different values of *R*_MM-B_. Generally, a CE with a larger amount of MMA results in a harder film which is fragile and can be easily detached from substrate^41^. The crack spacing and crack width as functions of *R*_MM-B_ are illustrated in Fig. 1c, suggesting an efficient strategy for cracking control via monomer mixing. The CE with an eclectic *R*_MM-B_ of 3–4 was chosen to acquire fine crackle templates.

In various dispersion systems, the crack pattern and crack size have been extensively described as functions of film thickness^41^. The coating thickness of a CE film also influences the resulting crackle template. We suggest that superior crackle templates contain channelling cracks dividing the plane into polygonal adjacent cells. The cracks should exhibit good connectivity and narrow crack width, but no detachment or delamination of the layer from the substrate. In this study, a large-scale spray coating system was used (Supplementary Fig. 1a), and the coating thickness was controlled by simply adjusting the Y stepping rate solely. Other parameters can also be easily adjusted to realize a certain CE coating thickness. The coating thickness of a CE film after drying shows a good reciprocal relationship with the Y stepping rate (see Supplementary Fig. 1b). The particle size distribution tests show that particles in CE2, CE3 and CE7 are mainly approximately 90 nm (Supplementary Fig. 2a), indicating a critical cracking thickness of ~760 nm (see Supplementary Note 2)^42^. The experimental results showed that the CE film would generate cracks after drying with a thickness larger than 900 nm (Supplementary Fig. 2b). Furthermore, experimental crackle templates of the CE3 with different thicknesses were obtained for comparison. As shown in Fig. 1d, the crack spacing and crack width both increase with increasing thickness of the coated CE film. Their increasing rates remain moderate at small thicknesses but suddenly increase when the thickness exceeds 3 μ m. The coated CE with a thickness of 2–3 μ m showed preferable crackle templates for fabricating high–quality CT-MM films. CT-MM films were further developed with crackle templates derived from different CEs, and they are termed CT-MM2, CT-MM3 and CT-MM7 for CE2, CE3 and CE7, respectively, with the same thickness of ~3 μ m. These samples are also further investigated in the following sections in terms of their optical transmissivity, EMI shielding performance and imaging quality (i.e., stray light distribution).

In addition, the large–scale coating process should be kept resistive and consistent because of the crucial influence of crack propagation speed on the cracking process^43^. Thus, the whole spray coating process is conducted in a nearly constant low–temperature and high–humidity environment. Specifically, the temperature in the spraying house is 18 °C and the humidity is 85% RH to slow down the evaporation rate of CE and facilitate the uniformly self-flowing flat of CE on large-scale and nonplanar surfaces. For the sake of comparison, crackle templates obtained by adopting CE3 with different drying conditions were tested. The CE was dried at such a constant and slow speed in the humid spraying house that it even did not generate cracks after thorough drying (Supplementary Fig. 3a), whereas the CE film cracked within minutes in the ambient environment and the cracked cells delaminated at the edges (Supplementary Fig. 3b). We then combined the advantages of the two strategies in our experiments and achieved a good control of CE drying and cracking, as shown in Fig. 1e. In this strategy, the drying time in the ambient environment (*t*_a_) plays an important role in the crack formation. Shorter values of *t*_a_ contributed to crack-cells with lighter detachment judging by the interference stripes at the cell edges, as depicted in Supplementary Fig. 3c–e. Moreover, the measured crack widths of crackle templates also show differences when treated with different *t*_a_, which is attributed to the slight scale effect of the warped crack sidewalls (Supplementary Fig. 3f). However, unreasonably short *t*_a_ will result in poor craquelures without connectivity, and a drying time *t*_a_ of ~5 min was found to be suitable for CE cracking (inset in Fig. 1e). Cracks are highly connected and show thoroughly penetrated channels to the bottom of the CE layer and break the CE into random polygonal cells, as seen in Supplementary Fig. 4, indicating perfect templates for junction-free metallic mesh film fabrication.

Therefore, the structural characteristics of the crackle templates can be adjusted triply. First, the cracking feature of CE can be tuned by adjusting the monomer mix proportion. Moreover, the crack spacing and crack width can be controlled by the coating thickness of the CE film. Third, the crack width can be modified by a drying-mediated strategy with tuneable cracking time. Based on the above, metallic structures of the finally produced CT-MM can also be controlled correspondingly. The finally obtained CT-MM shows disordered but highly connected metallic networks, with a metal line width of 0.5–2 μ m and a line spacing of 30–80 μ m (Supplementary Fig. 5). The SEM image shows the surface morphology of the coated CT-MM film (inset in Fig. 1a), and confirms the high quality of the metallic networks. These networks exhibit a junction-free property that is crucial to the film conductivity (see Supplementary Fig. 6a). Although metallic mesh spacing and structure orientation are quite different among networks, the line width and cell size are almost homogeneous throughout the whole CT-MM. Large-scale optical microscopy illustrates the excellent uniformity and high randomicity of metallic mesh structures (Supplementary Fig. 6b).

### Optical properties of the CT-MM film

**Figure 2:**
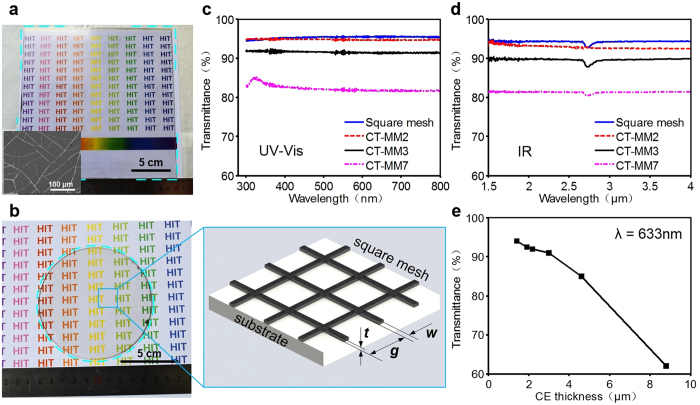
Optical images and light transmissivities of representative CT-MM films and square mesh. (**a**) Photograph of a fabricated large-area CT-MM film sample (200 ×  200 mm^2^). Inset: optical morphology image showing the metallic networks patterned on the substrate. (**b**) A typical square mesh sample (φ 100 mm) with magnified pattern geometry. The relative optical transmittance of CT-MM and square mesh films as a function of wavelength measured in the ranges of (**c**) 300–800 nm and (**d**) 1.5–4 μ m. Light transmissivity at λ  =  633 nm as a function of coated CE thickness.

A photograph of the obtained large-area CT-MM3 film is shown in Fig. 2a, which exhibits high transparency when taking a holoscopic view. Transparent CT-MM2 and CT-MM7 films are shown in Supplementary Fig. 7. As known, the transmittance of metallic mesh relies on the metal coverage rate over the whole film, which is easily evaluated via accessible structural parameters for regular meshes or grids. For example, the optical transmittance (*T*_*opt*_) of periodic square grids can be described as *T*_*opt*_ =  (1 − *w*/*g*)^2^, where *w* is the metal line width and *g* is the grid spacing^11^. However, this correlation cannot be applied to our unique CT-MM films obtained here because of the absence of a typical periodic structure; thus, experimental data were introduced to study their optical transmission performance. In comparison, a φ 10 mm regular metallic mesh sample with square silver meshes of *w* =  5 μ m, *g* =  250 μ m and Ag thickness of *t* ≈  200 nm was manufactured by a multi-step photolithographic process (Fig. 2b)^32^. The measured optical transmittance of the square mesh film is as high as 95.6%, which is quite similar to the calculated value of 96.0%. Relative optical transmission spectra of the as-obtained three CT-MM films show that the CT-MM films (e.g., CT-MM2) also exhibit wide and uniformly high transmittance in the UV-Vis-IR range of 300–4000 nm (Fig. 2c,d), indicating an ideal candidate for excellent IR-transparent material. Moreover, the optical transparency of the produced CT-MM film gradually becomes poor with increasing *R*_MM-B_ of CE. From the structural perspective, CT-MM2 possesses fine metallic networks with a line width at the sub-micron scale and line spacing of dozens of micrometres (Supplementary Fig. 7c). CT-MM2 shows a transmittance as high as ~95% in the UV-Vis spectrum. However, the plentiful fragmental metal wires on CT-MM2 prevent it from achieving higher transmittance. Moreover, the small mesh spacing resulted in a visible decrease of transmittance in the long wavelength region above 2 μ m, as shown in Fig. 2d. Although it possesses large-scale mesh spacing, CT-MM7 presents the most inferior transmittance, as low as 82%, among these films because it has the widest metal lines (see Supplementary Fig. 7b). CT-MM3 shows the preferable line width and line spacing allocation, and exhibits a relatively high transmittance of ~91% in the UV-Vis-IR spectrum.

In addition, the application of CT-MM film in optical/IR imaging systems depends closely on the high–order diffraction energy of the beam passing through the film. Specifically, stray light distribution of the film must be analysed for imaging quality evaluation. In fact, only the zeroth-order diffraction energy is beneficial for optical imaging, and all other higher orders increases the stray light level and degrades the imaging quality through the film. For the square mesh structures, a scalar diffraction theory can be applied to evaluate their transmission stray light distribution^11^. An optical point spread function (PSF) of square mesh grids is given to assess the ratio of the energy contained in each order of diffraction energy to the total incident energy (Supplementary Note 3). The transmission percentage of the zeroth-order beam (*T*_(0,0)_) can be described as 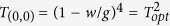. Thus, the square mesh sample presented above exhibits *T*_(0, 0)_ ≈  92.2% and a merited consequence of ~3.8% (fraction of the incident energy) in stray light. Moreover, this stray light energy will rapidly increase with increasing of duty ratio of metal meshes. Other high-order diffraction intensity can also be calculated using the PSF (Supplementary Fig. 8). However, there is a lack of a valid theory for analysing the stray light calculation of irregular metallic mesh films such as CT-MM.

**Figure 3:**
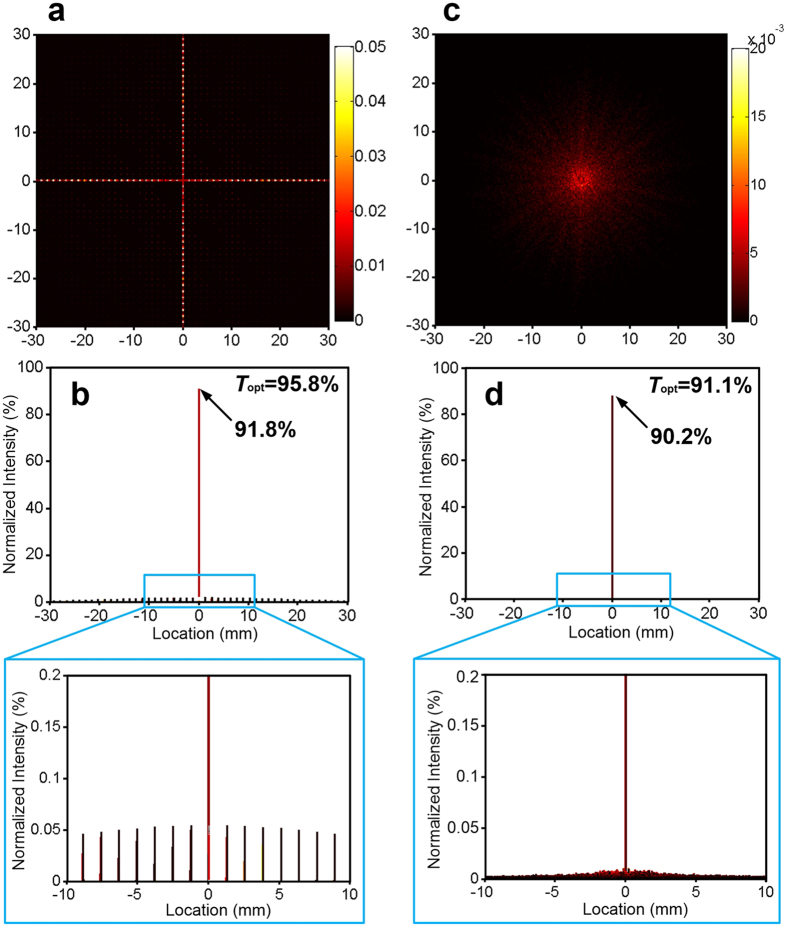
The simulated stray light distributions of square mesh and CT-MM. (**a**,**c**) Diffraction pattern spectrograms of (**a**) the square mesh sample (*g* =  250 μ m and *w* =  5 μ m) and (**c**) the CT-MM film (CT-MM3). (**b**,**d**) Normalized intensity spectra of diffraction spots related to the diffraction patterns and their partial enlarged drawings, respectively.

Here, we first analyse the stray light distribution of the mesh structure via a digital image processing approach based on Fourier optics. The stray light intensity through CT-MM film is further assessed by an experimental comparison method based on the diffraction of the regular structure. In the Fourier optics field, the intensity distribution of Fraunhofer diffraction of a screen can be calculated by its power spectrum (Supplementary Note 4). The metallic mesh film can be regarded as a ‘black and white’ screen in the optical field, with perfect reflection in the metallic region and transmission in the hollow region. Fourier calculation can be conducted using a monochrome image with black line meshes, and a diffraction pattern can be obtained from the frequency spectrum of the screen image. Figure 3 demonstrates the calculated diffraction patterns of a square mesh sample and a CT-MM film. The calculation area is 2 ×  2 mm^2^. The diffraction pattern of the square mesh sample is an orthogonally distributed 2-D ‘cross’ spots array (Fig. 3a,b). The optical transmittance is calculated at approximately 95.8%, and the intensity of the zeroth-order spot is ~91.8%, which are quite similar to the expected values (i.e., *T*_*opt*_ =  96.0%, *T*_(0, 0)_ ≈  92.2%) as the PSF predicted. A series of dispersed high-order diffraction spots with a relative intensity of ~0.05% surround the main spot. These diffraction spots will cause a disturbance to the image sensors behind the film, because they produce deceptive objects when imaging and reduce the contrast of the image^11^. In contrast, the CT-MM film exhibits very weak diffraction characteristics. There are no maximum high-order spots in the diffraction pattern; only a slight and uniform enhancement in the background region can be observed (Fig. 3c,d). Transmission light energy through CT-MM film is almost all centred on the zeroth-order spot, which shows a normalized intensity of 90.2% of the total 91.1% transmitted energy. This intensity means that less than 1% optical energy is dissipated by the CT-MM structure diffraction. Therefore, the stay light intensity of a CT-MM is approximately one–quarter that of the conventional square mesh. In other words, the optical signal-to-noise ratio (SNR) through the CT-MM (~100) is approximately three times larger than that through the square mesh (~23) when applied in imaging, indicating a great improvement in the imaging quality and reliability of optical sensors (see Supplementary Note 5). Notably, CT-MM films with wide metallic wires and narrow mesh cells will present enhancement in the stray light.

**Figure 4:**
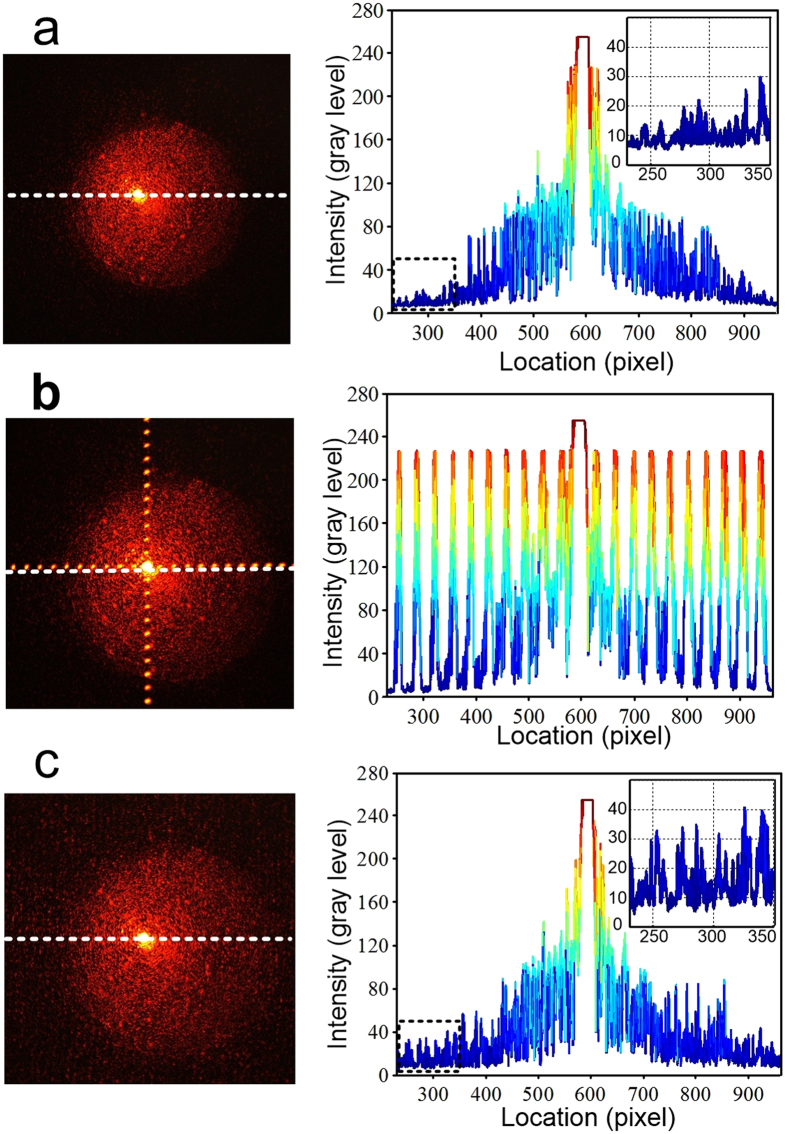
Stray light distribution testing with different samples. Detected diffraction patterns with digital grey level graphs (along dash lines) of (**a**) a bare quartz, (**b**) a square mesh/quartz sample and (**c**) the CT-MM3/quartz sample. Insets in (**a**,**c**) are the enlarged grey level graphs at high–order regions.

Furthermore, a detection set-up was established to efficiently evaluate the stray light distribution of CT-MM films (see Supplementary Fig. 9). Concerning the large diffraction differences between the two types of samples, the regulated incident beam intensity must satisfy the requirement that the strongest high-order diffraction spot among all samples does not saturate the CCD sensor and all diffraction spots exhibit maximum grey levels in the digital image, simultaneously. Different diffraction patterns were obtained when the incident light was transmitted through these samples, as demonstrated in Fig. 4b,c. Obviously, zeroth-order spots of diffraction patterns are all saturated as expected, and we are mainly concerned with the high-order diffraction (i.e., stray light) distributions. Light transmitted through the bare quartz shows no diffraction and exhibits a highly centred transmission pattern with faint background light noises (Fig. 4b). The grey level of light noises is approximately 10 in the digital image. Regarding the square mesh sample, its diffraction pattern is similar to the calculated results in Fig. 3a, and the scattered high–order spots show a uniformly high grey level (~228) seen from the digital energy distribution (Fig. 4c). However, highly homogeneous stray light distribution can be observed in the tested diffraction pattern of CT-MM3, where grey levels exhibit an average increment of ~15 compared with the pattern of bare quartz. Specifically, the first-order diffraction spot of the square mesh sample possesses a transmitted energy of ~0.05% (see Fig. 3b and Supplementary Fig. 8), and it exhibits a corresponding grey level of 218 in the digital image as detected. Thus, a 15 grey–level increment in the detected diffraction image of CT-MM3 film represents a corresponding energy enhancement of ~0.003% in stray light in the diffraction pattern of CT-MM3. Moreover, stray light energy dissipation of CT-MM2 was calculated to be as low as ~0.5%, and the value of CT-MM7 increased to 2.2% (Supplementary Fig. 10c). Experimental grey level increments in stray light of CT-MM2 and CT-MM7 of 8 and 40 are tested, respectively, as shown in Supplementary Fig. 10a,b,d. Consequently, the CT-MM films generate only uniformly distributed and faint stray light after light striking, and they do not bring any image degradation to the sensors, even in a harsh optical environment, when applied in transparent EMI shielding windows of high-performance optoelectronic systems. Moreover, the optical haze, or diffuse scattering, of CT-MM samples was further measured with a UV-Vis spectrometer with an integrating sphere. The optical haze for the CT-MM3 is very small (~2% at 550 nm), as depicted in Supplementary Fig. 11. The optical hazes for CT-MM2 and CT-MM7 are ~1% and ~5%, respectively at 550 nm. These values indicate that CT-MM films have good optical properties.

### EMI shielding performance of the CT-MM film

The EMI shielding performance of materials is evaluated by the EMI SE, which is defined as the logarithmic ratio of incident EMI power (*P*_*i*_) to transmitted EMI power (*P*_*o*_); that is, SE(dB) =  −10 log(*P*_*o*_/*P*_*i*_) =  − 10 log(*T*_*EMI*_)^5^,^8^,^10^. Obviously, the higher the dB level of SE is, the fewer EM waves are transmitted through the material. For a metallic mesh film, the EM wave is shielded by dominantly reflecting it back to the surrounding space, and its EMI SE is largely influenced by the conductivity of the film^30^. It is well known that the conductivity of metallic mesh films also depends on the geometrical structure of metal networks. Wider, thicker, and denser metallic networks exhibit better conductivity and EMI shielding performance but may lower the film’s optical transparency as discussed above. Because highly transparent materials are usually followed by modest EMI shielding performance and strong EMI shielding materials are often accompanied by inferior optical transparency (known as the inherent conflict between optical transmittance and EMI SE)^6^,^7^,^8^,^9^,^10^,^21^, it is intuitive to compare the transmittance and EMI SE of reported films for better understanding of the rational design of transparent shielding CT-MM applied in optical windows or domes as sensor or detector components.

**Figure 5:**
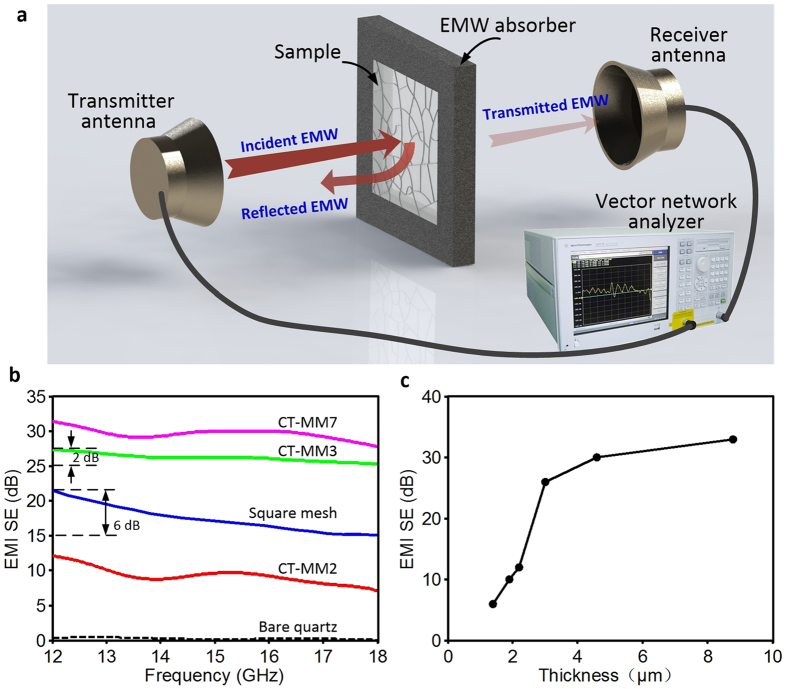
Microwave shielding performances of CT-MM films and the testing schematic. (**a**) Schematic illustration of the EMI shielding measurement setup. The sample was installed in a centre-hollowed EMW absorber (foam, 500 ×  500 ×  5 mm^3^). (**b**) EMI SE of CT-MM films measured in the frequency range of 12–18 GHz. (**c**) EMI SE at a frequency of 18 GHz of CT-MM films fabricated by CE3 with different thicknesses.

The EMI SE of CT-MM films was measured in the frequency range of 12–18 GHz (Ku-band) by a horn antenna set-up composed of a vector network analyser, transmitter antenna and receiver antenna (Fig. 5a). The measurement was calibrated with a bare quartz substrate. Figure 5b shows the EMI SE spectra of transparent CT-MM films fabricated with different CEs. As we can see, the bare quartz is obviously transparent to EM waves and exhibits almost no shielding ability. In contrast, the CT-MM films show strong EMI shielding ability even with high optical transparency. The EMI SE of the CT-MM3 film, which exhibits an optical transmittance of ~91%, is measured to be ~26 dB (i.e., power attenuation of ~99.7%) in the Ku-band frequency range, indicating that only 0.3% of the incident EMI power can be transmitted through the film. A stronger shielding effect would be achieved at a lower frequency range, which is attributed to the increasing reflection of longer-wavelength microwaves from CT-MM films^31^. Moreover, the least transparent CT-MM7 film showed the best EMI shielding performance with an SE value as high as ~30 dB (i.e., power attenuation of ~99.9%). It is noteworthy that the CT-MM2 film exhibits a low EMI SE of only ~10 dB, whereas the most transparent square mesh sample possesses much higher SE values of 15–21 dB at 12–18 GHz. This outcome is probably a consequence of poor connectivity between metal wires on the CT-MM2 film. We can easily find that the regular metallic grids on the square mesh sample are highly connected; but relatively, many fragmental metal wires on CT-MM2 film show no connection with metallic networks. However, few fragmental metal wires were observed in CT-MM3 and CT-MM7. It can be indicated that the EMI SE of the CT-MM films was mainly contributed by the conductivity rather than the pure coverage rate of metallic meshes, which may directly influence optical transparency. Thus, the fragmental wires are inversely correlated with both the film transparency and the EMI shielding performance of CT-MM film, and they should be eliminated in the template processing stage.

In addition, the SE curves of CT-MM films are more stable than those of the square mesh sample, as shown in Fig. 5b. For example, the SE value of CT-MM3 film exhibits a small decline of only ~2 dB from 12 GHz to 18 GHz, whereas the corresponding reduction of the square mesh film is almost 6 dB, which is much larger. Good and uniform EMI shielding performance in the high–frequency microwave range is crucial in military and space applications. It is quite difficult to obtain a stably high SE in one shielding film without sacrificing the optical transmittance, especially the IR transmittance. We believe that the small mesh spacing of CT-MM contributes to its high and stable EMI SE in the high–frequency microwave region. The unique mesh structures in the CT-MM film derived an excellent EMI shielding effect and decent optical transparency simultaneously. Moreover, the EMI shielding performance of the CT-MM films fabricated by CE3 with different thicknesses was also tested, as shown in Fig. 5c. With increasing CE thickness, the EMI SE at 18 GHz of CT-MM film first increases sharply and then slows to a milder rate at the thickness of ~3 μ m. CT-MM films prepared by thin CE3 with thickness less than 3 μ m exhibit relatively fragmental metal wires that negatively influence the conductivity and lead to much poorer EMI SE. However, much thicker CE results in less transparent CT-MM films that are unsuitable for high-quality optical windows or domes because of the improper metal coverage. Therefore, a rational CE thickness must be achieved for crackle templates in the CT-MM processing. Strategies such as CE optimization, thickness control, and temperature/humidity adjustment can further be taken to realize modification of crackle templates and the resulting CT-MM films.

### Dome-shaped transparent EMI shielding windows

**Figure 6:**
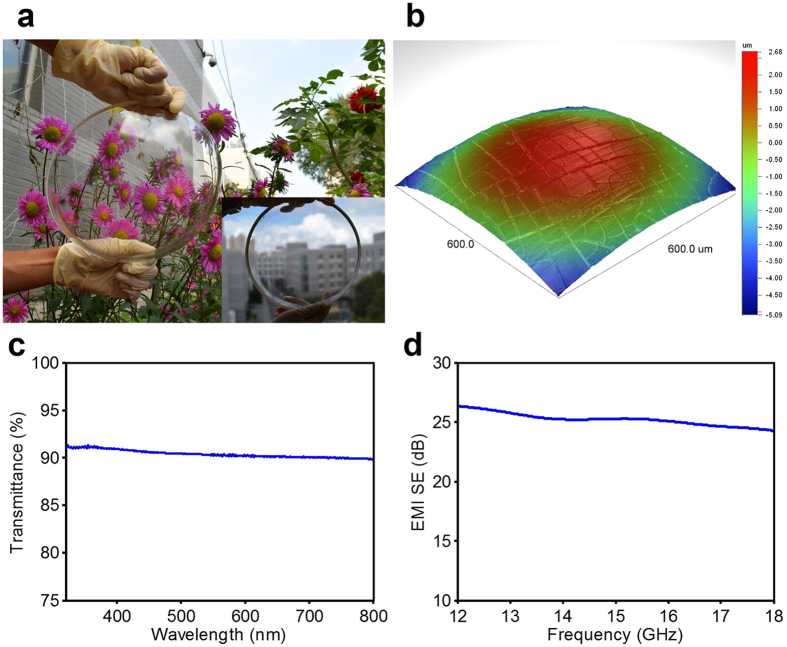
Photographs and performances of a large-calibre CT-MM dome. (**a**) A photograph and (**b**) surface profile of a large-calibre quartz dome coated with CT-MM film. The dome is a spherical cap with an outer diameter of 220 mm, a cap height of 85 mm and a wall thickness of 7 mm. The inset shows a macrophotograph of the CT-MM dome. (**c**) The optical transmittance spectrum in 300–800 nm regions and (**d**) the EMI SE in the Ku-band of the CT-MM dome, respectively.

Owing to its distinctive fabrication process, CT-MM also shows superiority to other transparent EMI shielding materials in terms of the processibility for non-planar substrates. Using the aforementioned spray coating method and modifying the CE coating thickness (because of the distinctive self-flowing effect on the curved surface), we have prepared CT-MM film on a large-calibre quartz dome, as shown in Fig. 6a,b. This complex–shaped CT-MM dome also shows high optical transparency and clarity, good EMI shielding performance and excellent imaging quality (Fig. 6c,d). Such a CT-MM dome can be applied in complex optoelectronic systems, such as aerial cameras and aircraft cockpits. Dome substrates with a curvature radius larger than 20 mm are deemed suitable for the proposed fabrication technique of CT-MM. Moreover, hybrid complex-shaped CT-MM could also be fabricated on substrates with multiple curvatures (e.g., wave-shaped glass), using optimized CE and a modified spray coating technique, which is in progress.

## Discussion

We have developed large-area metallic mesh films on multi-shaped substrates based on crackle templates that were fabricated by spray–coated crackle emulsions and a controlled drying process. Broadband high optical transmittance, strong EMI shielding effectiveness and very low stray light interference can be obtained. The metallic mesh can realize high transmittance of 91% in the UV-Vis-IR spectrum and strong EMI shielding of 26 dB in the Ku-band frequency range. More importantly, its specific stray light in transmission direction shows an increment as low as only ~1% to the system and realizs a high optical SNR of 100, which far surpasses the best values of regular metallic grids or rings. The excellent processing scalability and cost-saving procedure of this metallic mesh film also provides it with huge potential for practical applications as a high-performance transparent EMI shielding material in areas such as aerospace, medical facilities and next-generation EM–compatible optoelectronic facilities.

## Methods

### Materials

Reagent grade methylmethacrylate (MMA), butyl acrylate (BA), acrylic acid (AA) and hydroxyl propyl acrylate (HPA) as monomers, ammonium peroxodisulfate (APS) as an initiator, OP-10 as a non-ionic emulsifier, sodium bicarbonate (NaHCO_3_) as a buffer and propylene glycol methyl ether acetate (PGMEA) as a remover were obtained from Aladdin Reagent Co. Ltd. (Shanghai, China). Sodium dodecyl sulfonate (SDS) as an anionic emulsifier was purchased from Sigma-Aldrich Corporation (St. Louis, Mo USA). An aqueous solution of 25% ammonia as a neutralizer was supplied by Tianjin Fuyu Fine Chemical Co., Ltd. (Tianjin, China). All reagents were used as received, without further purification.

### Preparation of crackle emulsion

Taking the preparation of CE3 (*R*_MM-B_ =  3) for example, a monomer mixture was first obtained by commixing 30.0 g of MMA, 10.0 g of BA, 1.5 g of AA and 3.0 g of HPA in a beaker; meanwhile, 0.2 g of APS was dispensed in 20.0 g of ultrapure water to obtain the initiator solution. Next, an aqueous solution dissolved 0.8 g of SDS and 1.2 g of OP-10 in 35.0 g of ultrapure water, 10 wt% monomer mixture and 0.5 g of NaHCO_3_ were emulsified at 1000 rmp using a mixer to prepare an emulsified mixture. The emulsified mixture was heated to 75 °C, and 20 wt% initiator solution was added with continuously blending. This mixture was kept at 80 °C for 30 min, and both the remaining monomer mixture of 90 wt% and initiator solution of 80 wt% were continuously dropped in with stirring at 80 °C for 3 h. The reaction mixture was then kept at 85 °C for 2 h. After cooling, 1.0 g aqueous ammonia solution was added to the reaction mixture for neutralization. The prepared CE3 was finally gained after filtrating with a filter cloth (500 ×  500 meshes per inch) 3 times.

### Fabrication of CT-MM film

The prepared CE was first deposited on the cleaned quartz by spray coating using a homemade large–scale spraying system. The system comprises a uniform air spray module, a dual-axis motion module, and a temperature/humidity regulator in a house. The processing environment in the spraying house was maintained constant with a temperature (*T*) of 18 °C and a relative humidity (*H*_R_) of 85%. The CE solution was injected by an automatic syringe pump (Ristron, China) at a speed of 2 mL/min. The nitrogen gas pressure for air spraying was kept at 0.03 MPa through a pressure stabilizion valve (SMC, Japan). The spray nozzle (Spraying Systems Co., USA) is installed approximately 15 cm above the substrate and moves in the ‘zig-zag’ mode; with reciprocation in the X direction (120 mm/s) and step motion in the Y direction (rate adjustable at integral steps). Afterwards, the coated sample was left self-flowing flat in the spraying house for 10–30 min, and then transferred to the ambient environment (*T* =  22 °C, *H*_R_ =  55%) drying and cracking for a controlled period (time adaptable), and finally transferred back to the spraying house for thorough drying. Stereotyped crackle template was gained after drying for ~20 h. Next, a conductive silver (Ag) film with a thickness of 200 nm was deposited on the crackle template using an e-beam evaporator (Technol Co., China) at room temperature (with base vacuum of 4 ×  10^−4^ Pa, accelerating voltage of 9 kV and beam current of 240 mA). Finally, lift-off was conducted by soaking and sonicating the sample for 2–3 min in the CE remover, and a CT-MM sample was obtained.

### Characterization method

Particle size distributions of CEs were measured by a dynamic light scattering instrument (Zetasizer Nano-S, Malvern Instruments Ltd., UK), all with sample volumes of ~2 mL. The thickness of coated CE films was measured using a Bruker Dimension Icon scanning probe microscope (SPM) after thoroughly drying for 2 h. SEM observation was performed with a Zeiss Sigma field emission scanning electron microscope. The morphology was investigated on planar samples by an SPM and on nonplanar samples by a Dektak 150 surface profiler (Veeco, USA). Optical photographs and images were obtained using a Nikon D3300 DSLR camera and a Nikon SMZ1500 stereomicroscope. The transmittance of samples was measured in the UV-Vis spectrum region using a UV-3101PC spectrophotometer (Shimadzu, Japan) at wave–lengths of 300 nm to 800 nm with scanning steps of 0.5 nm and in the near–IR spectrum region using a Nicolet 8700 FT-IR spectrophotometer (Thermo Electron, USA) at wave–numbers of 7400 cm^−1^ to 2500 cm^−1^ with scanning steps of 7.7 cm^−1^. The EMI SE was measured in the Ku-band frequency range of 12–18 GHz using a horn antenna system composed of an Agilent E8363B PNA series vector network analyser, a transmitter antenna, and a receiver antenna in an anechoic chamber. The sample was homocentrically installed in the central position between the transmitter and receiver antennas, with the same distance of 0.5 m. Stray light distributions were detected by a set-up comprising a laser source (Melles Griot, USA), collimation and expansion components, attenuation slices, a sample holder, a focusing lens (*f* ≈  50 mm), CCD sensor (Daheng Co., Ltd., China) and a laptop computer. All measurements were tested under ambient atmospheric conditions. Grey level graphs of diffraction patterns were obtained by converting the detected bitmap files to greyscale images and extracting their grey level distribution with MATLAB. The optical haze was measured with a PerkinElmer Lambda 750 UV-Vis Spectrometer with a Labsphere RSA 60 mm integrating sphere.

## Additional Information

**How to cite this article**: Han, Y. *et al.* Crackle template based metallic mesh with highly homogeneous light transmission for high-performance transparent EMI shielding. *Sci. Rep.* **6**, 25601; doi: 10.1038/srep25601 (2016).

## Supplementary Material

Supplementary Information
