# Supplementary material for: Crackle template based metallic mesh with highly homogeneous light transmission for high-performance transparent EMI shielding
Source: Sci Rep. 2016 May 6;6:25601. doi: 10.1038/srep25601 (PMC4858675; doi:10.1038/srep25601)
Supplement: Supplementary Information [file srep25601-s1.pdf]

Supporting Information

Crackle template based metallic mesh with highly homogeneous light transmission for high-performance transparent EMI shielding

Yu Han[1†], Jie Lin[1†], Yuxuan Liu[2], Hao Fu[1], Yuan Ma[3], Peng Jin[1]* and Jiubin Tan[1]*

[1] Center of Ultra-precision Optoelectronic Instrument, Harbin Institute of Technology, Harbin 150080, P. R. China

[2] School of Energy Science and Engineering, Harbin Institute of Technology, Harbin 150001, P. R. China

[3] Department of Electrical and Computer Engineering, Dalhousie University, Halifax, NS B3H 4R2, Canada

[†] These authors contributed equally to this work.

* Correspondence and requests for materials should be addressed to P.J. (P.Jin@hit.edu.cn) and J.T. (jbtan@hit.edu.cn).

Supplementary Figures

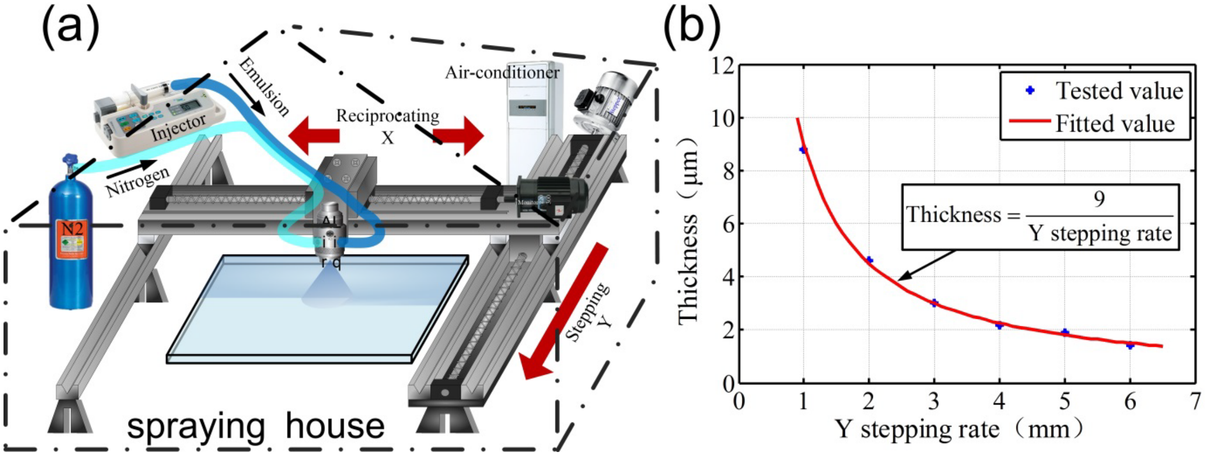

Supplementary Figure 1 | Large-scale spray coating system and coating thickness curve. (**a**) Schematic of the spraying system used for large scale CE coating. Crackle templates were obtained by spray coating CE3 on flat substrates with different Y stepping rates and a fixed injection rate of 2 mL/min, a X reciprocating speed of 120 mm/s and an air pressure of 0.03 MPa. (**b**) Thickness variation in dried crackled templates versus the Y stepping rates. The least-squares fitting curve and equation for the tested values are shown in the figure.

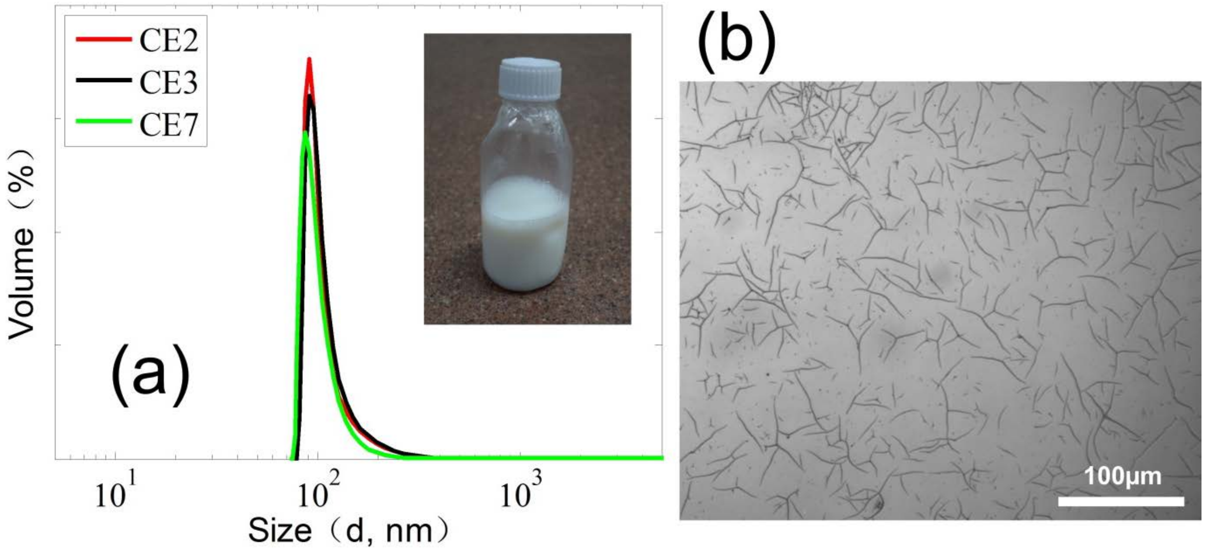

Supplementary Figure 2 | Particle size and cracking property of synthesized CE. (**a**) Particle size distributions of the synthesized CEs. Inset is a bottle of CE sample. (**b**) Optical microscopic image of isolated cracks generated on a 920 nm-thick CE3 layer.

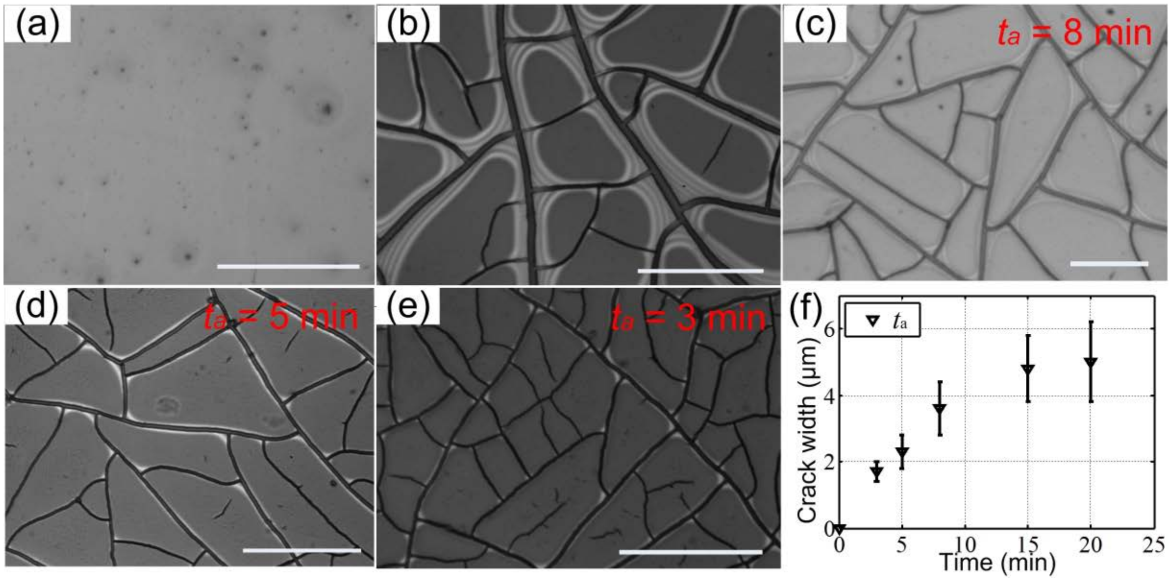

Supplementary Figure 3 | Influences of the different drying-mediated processes on resulted crackle template. Optical microscopic images of templates formed in CE3 layer (thickness of ~3 μm) when dried (**a**) in the spraying house for 22 h; (**b**) in the ambient for 22 h; and with combined strategies that the coated samples were first left in the spraying house for 20 min, then transferred to the ambient cracking for (**c**) t_a = 8 min, (**d**) t_a =5 min and (**e**) t_a =3 min, respectively, and finally transferred back to the spraying house drying for 20 h. Scale bars: 50 μm. (**f**) A plot of average crack width versus the cracking time t_a.

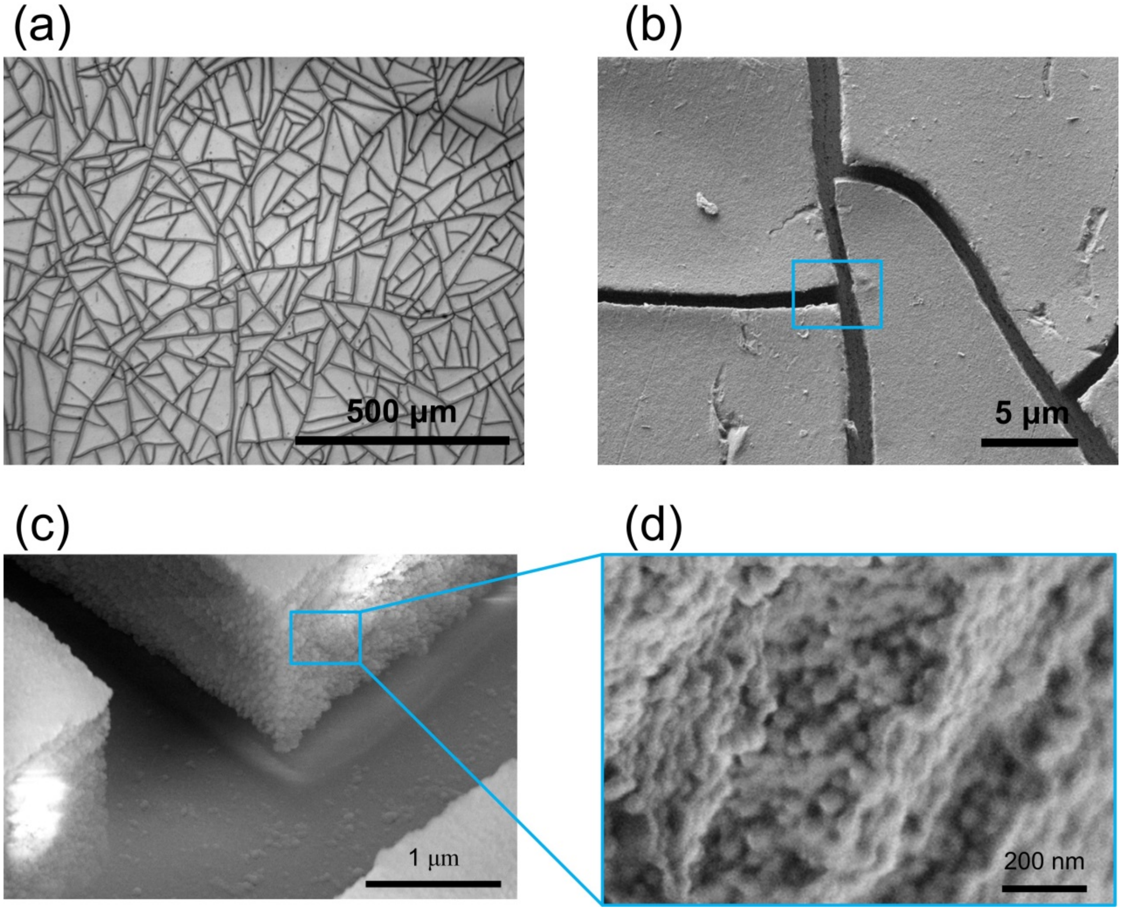

Supplementary Figure 4 | Crackle template fabricated using CE3. (**a**) Optical microscopic image showing a large-scale network of cracks in a CE3 layer dried with t_a = 5min. SEM images of (**b**) several interconnected cracking channels, (**c**) a magnified crack junction taken at a 60° angle of inclination and (**d**) the dried particles on the side-wall of the crackle.

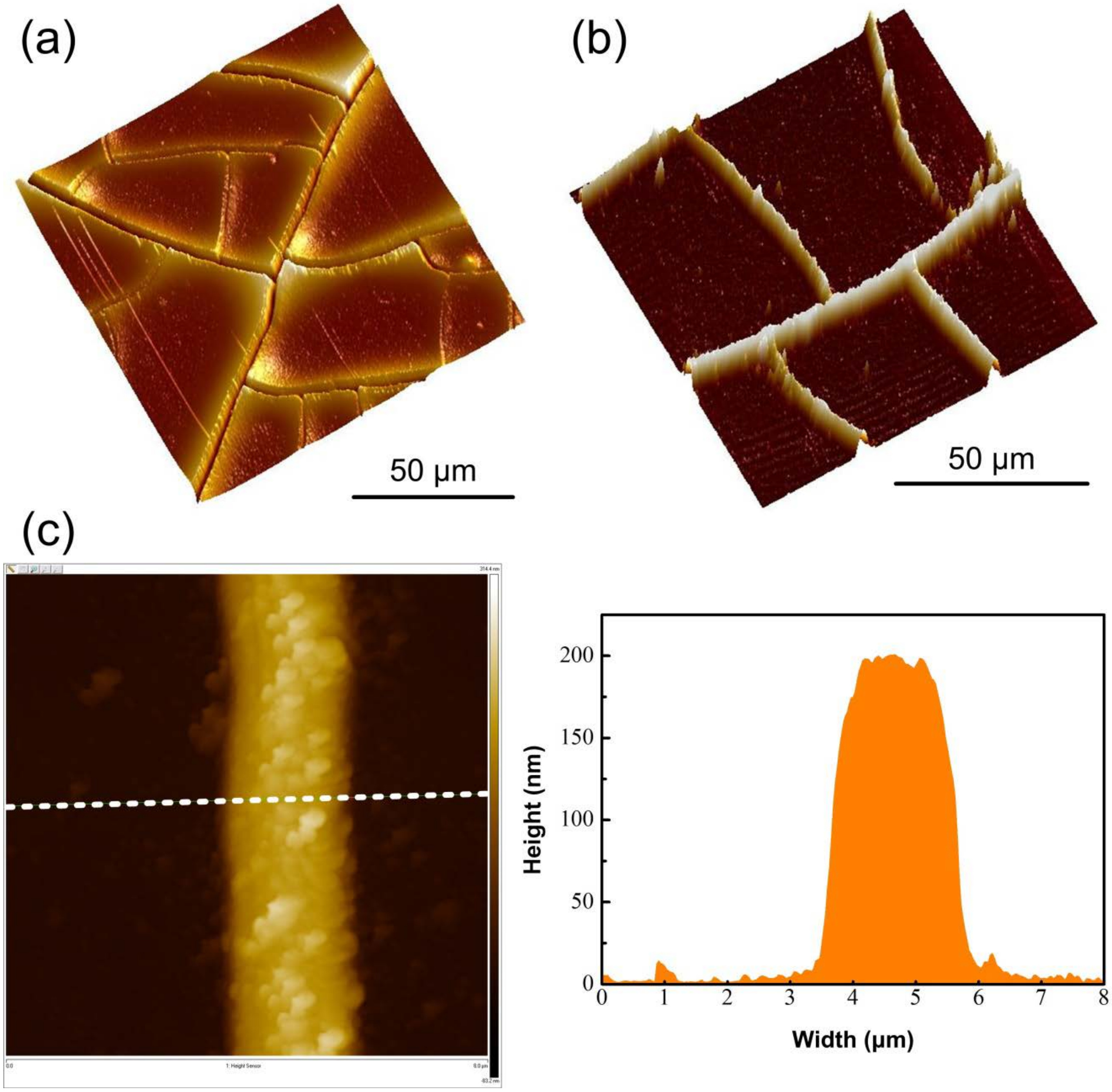

Supplementary Figure 5 | AFM characterization. AFM images of (**a**) a crackle template and (**b**) the CT-MM structure. (**c**) AFM topography image and the cross profile of a metallic line of the CT-MM film showing a metal line width of about 2 μm and a line height of 200 nm.

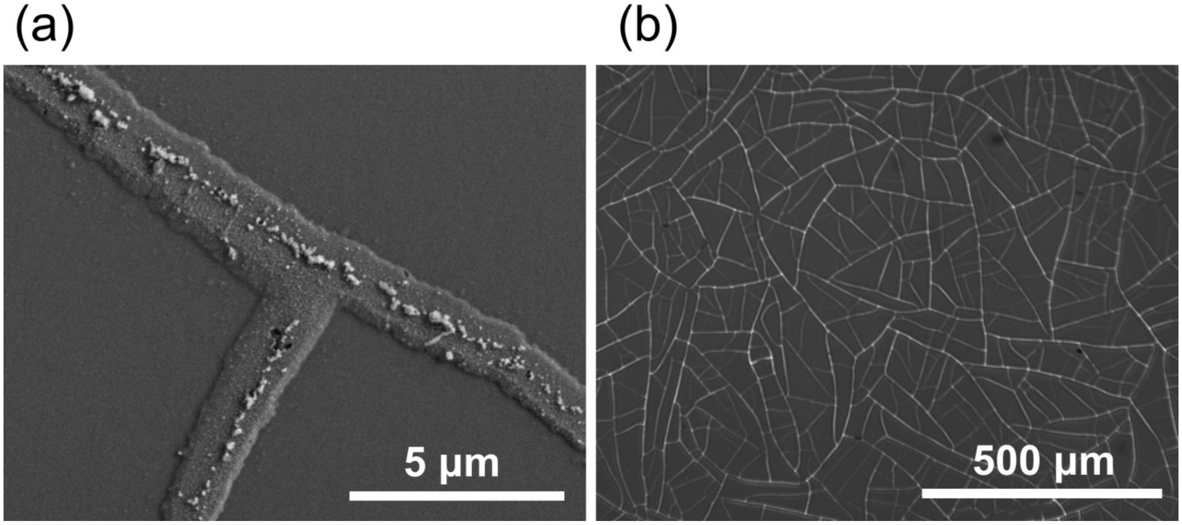

Supplementary Figure 6 | Metallic mesh images. (**a**) SEM image of a metallic mesh junction within the network of the CT-MM film. (**b**) Optical microscopic image of the finally obtained CT-MM film. The observed area is 1200 ×1200 μm^2.

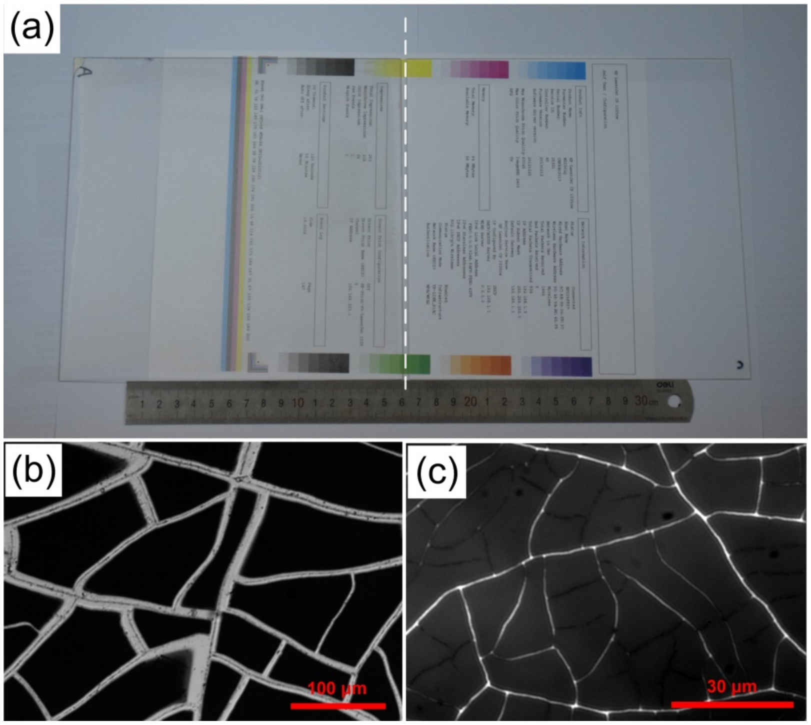

Supplementary Figure 7 | Photograph and micrograph of CT-MM7 and CT-MM2. (**a**) Photograph of a CT-MM7 (left) and a CT-MM2 (right) sample against a white background with black words. Optical micrographs of the magnified metallic networks within (**b**) CT-MM7 and (**c**) CT-MM2 films, respectively.

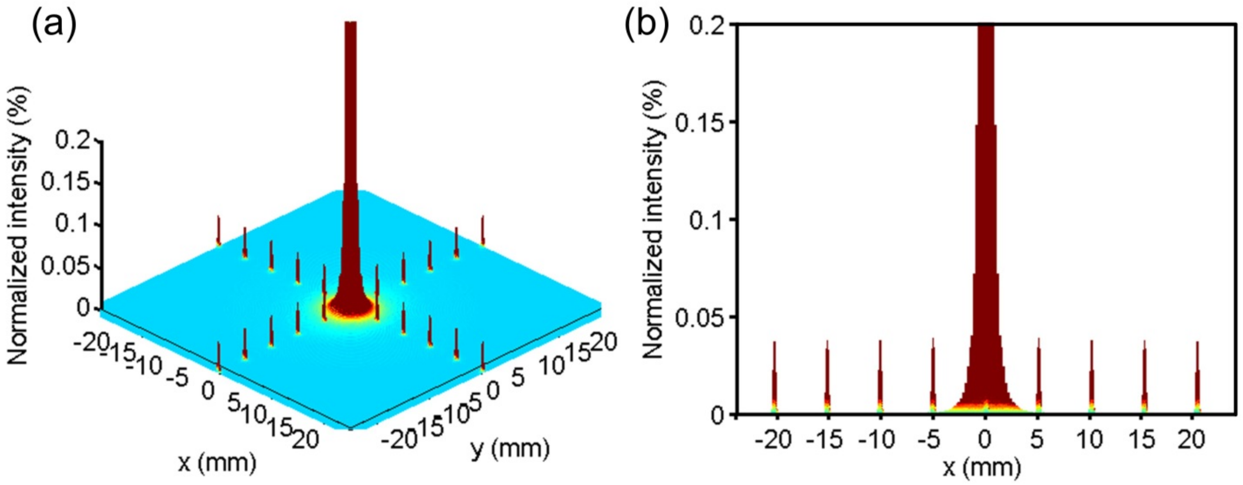

Supplementary Figure 8 | Calculated diffraction results. Calculated (**a**) diffraction pattern and (**b**) spots intensity of the square mesh sample by using PSF in Supplementary Note.3.

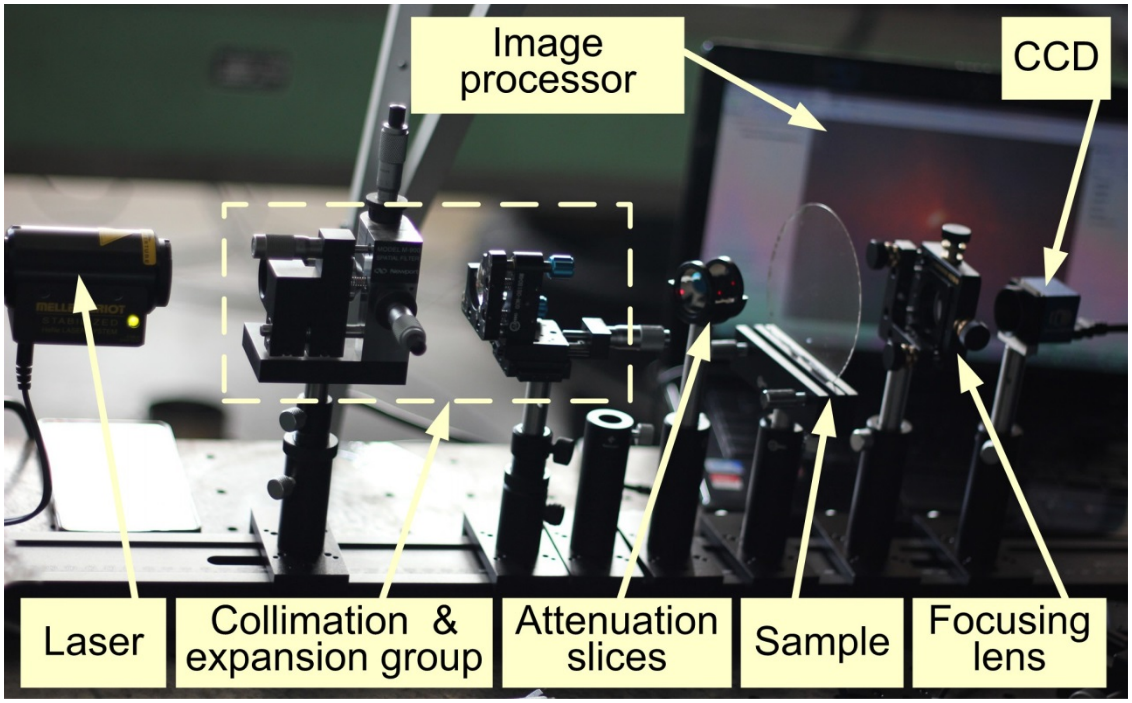

Supplementary Figure 9 | Experimental setup for transmission light detection. The incident beam was collimated and expanded to ~5 mm in diameter, and the incident intensity was regulated by a group of attenuation slices and was kept constant during testing.

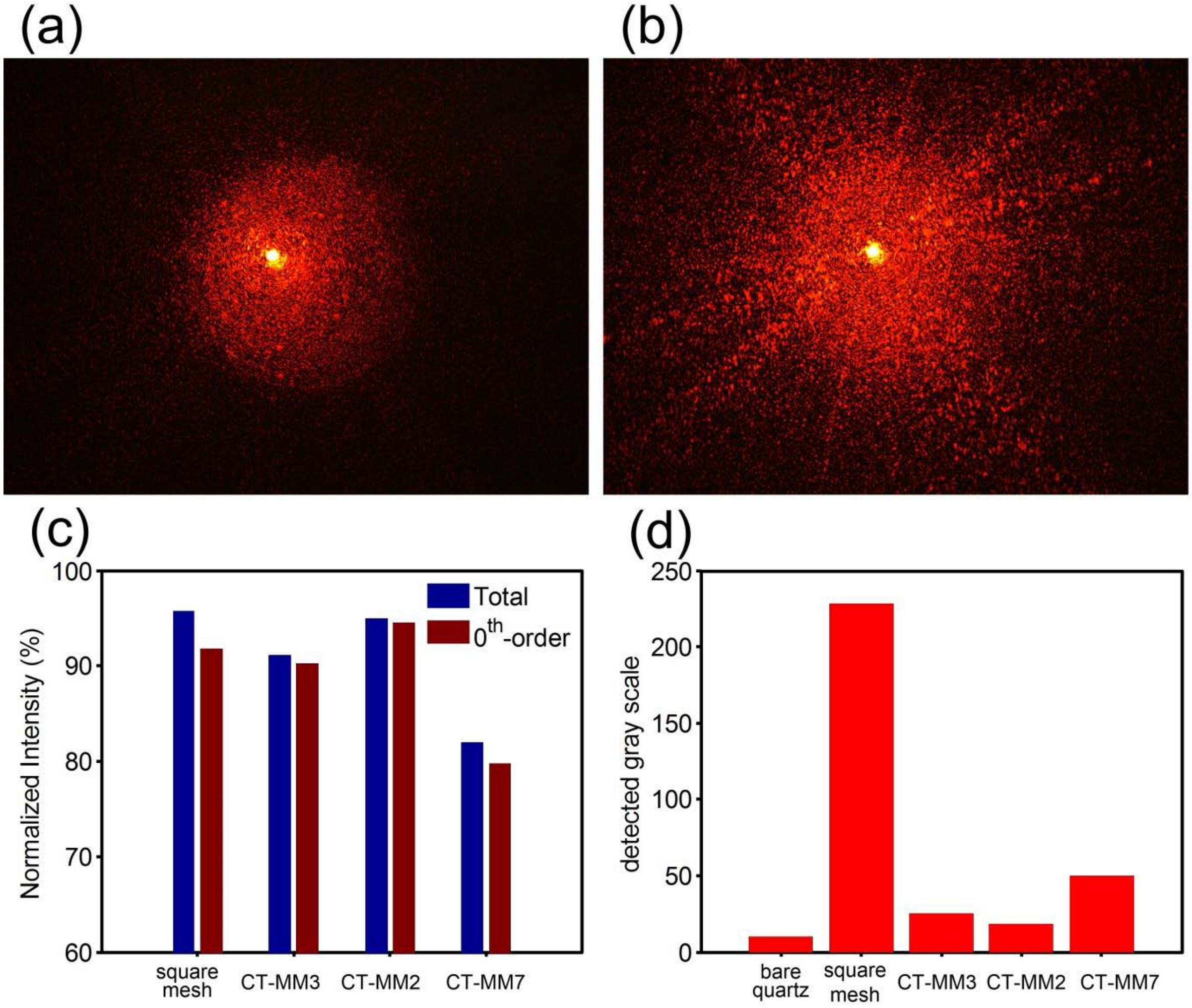

Supplementary Figure 10 | Experimental diffraction results. Detected diffraction patterns of (**a**) the CT-MM2/quartz sample and (**b**) the CT-MM7/quartz sample. (**c**) Calculated total transmittance and transmittance in 0th-order of different samples. (**d**) Grey levels at high order regions in detected diffraction patterns of different samples.

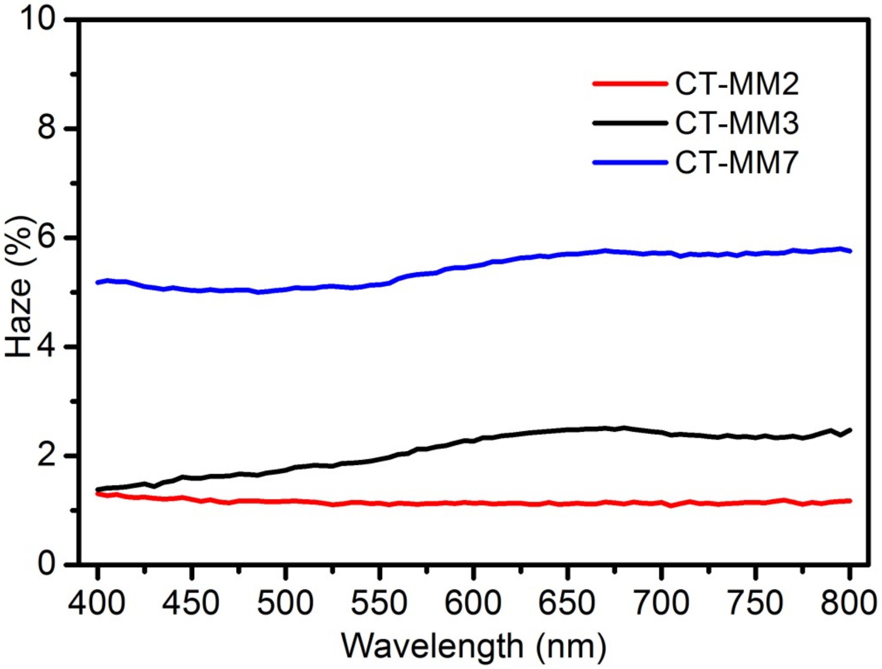

Supplementary Figure 11 | The optical haze of CT-MM films. Plots of the haze versus wavelength for CT-MM2 (red line), CT-MM3 (black line), and CT-MM7 (blue line), showing that the CT-MM films with good optical properties. The mechanism of haze measurements can refer to Supplementary Ref. 1.

Supplementary Notes

Supplementary Note 1. Preparation of crackle emulsion

At present, researchers use a commercial nail for self-forming crackle templates. A relatively easier dissolving method can be applied to remove the sacrificial template at the last step compared with other approaches such as using the TiO_2 gel.[2, 3] However, the unknown components or fillers within the commercial nail prevent us from getting metallic networks with good processibility, transmisivity and conductivity. An ideal emulsion should perform a homogeneous dispersion with specific rheological behavior and exhibit cracking property. It requires dispersion with reasonable viscosity (to facilitate the spreading of CE on large-scale) and suitable mixing ratio between hard monomers and soft monomers (to minimize the buckling of cracked cells), as well as rational coating technique that can avoid thickness non-uniformity and slowdown secondary flows induced by surface tension. Moreover, good drying strategy is also needed for cracking control.

Crackle-emulsion (CE) used in this research is a water-based acrylic dispersion, and it was synthesized by a traditional emulsion polymerization method.[4] In the CE preparation, AA and HPA were introduced as functional monomers to enhance the adhesivity and water solubility of CE. However, excessive AA and HPA would increase viscosity and particle size after polymerization, even cause large number of sediments that negatively influence the quality of CE. On the other hand, the amounts of emulsifier and initiator are also very important. Deficient amount of emulsifier or initiator will result in insufficient polymerization of monomers, while excessive emulsifier and initiator lead to fast polymerization; both situations produce poor CE. Here, 3.4 wt% of AA, 6.7 wt% of HPA, 5.0 wt% of emulsifier and 0.5 wt% of initiator to the total amount of monomers were selected, and the emulsifier composed of 3 part of OP-10 and 2

part of SDS was preferred. More importantly, the primary amount of hard monomer (MMA) and soft monomer (BA) essentially determined features of the CE solution. The total amount of monomers was adopted according to the final solid content needed, and a solid content of 40% of CE was selected for spray coating in our experiments. Taking CE3 ($R_{\text{MM-B}}$ =3) for example, specific ingredients for the preparation of CE3 are listed in Supplementary Table 1. Proportioning ratio of MMA and BA ($R_{\text{MM-B}}$) was tuned from 1 to 7 as designed. Monomer dosages of CE with different values of $R_{\text{MM-B}}$ are listed in Supplementary Table 2.

Supplementary Note 2. Critical cracking thickness analysis

When a thin film of wet coating containing suspended colloidal particles is dried on a substrate, evaporation of the solvent concentrates the particles into a closed packed array. The film generally binds to the substrate and resists deformation in the transverse direction giving rise to transverse tensile stresses.[5] If the particles are hard, the film cracks to release the stresses. It has long been observed that the maximum crack-free thickness (i.e., critical thickness) for a film plays an important role in cracking analysis. The critical stress for cracking can be presented by the following equation[6]:

$$h_c = 0.64\left[\frac{GM\phi_{rcp}R^3}{2\gamma}\right]^{1/2}\left[\frac{2\gamma}{(-P_{\max})R}\right]^{3/2} \quad (1)$$

Here, R is the particle radius, γ is the solvent-air interfacial tension, G is the shear modulus of the particles, M is the coordination number, φ_{rcp} is the particle volume fraction at random close packing, and $P_{\max}$ is the maximum attainable capillary pressure. The dimensionless capillary

pressure was determined as $(-P_{\max})R/2\gamma = 1.2 \pm 0.08$ using Kelvin equation[5]. Choosing a value of 1.15, the critical thickness can be reduced as:

$$h_c = 0.52\left[\frac{GM\phi_{rcp}R^3}{2\gamma}\right]^{1/2} \tag{2}$$

With *G*=0.8 GPa, *M*=7.0, $\phi_{rcp} = 0.68$, *γ*=0.072 N/m and *R* = 90 nm, a critical thickness of 710 nm can be obtained. Moreover, the critical thickness for cracking increases with the size of colloidal particles.

Supplementary Note 3. Diffraction Calculation based on PSF

The diffraction characteristics of a regular metallic mesh structure were analyzed using a pupil function. The pupil function of a circular aperture square mesh can be expressed as the following equation:

$$t_s(x,y) = circ\left(\frac{\sqrt{x^2+y^2}}{Ng/2}\right)\left\{\left[rect\left(\frac{x}{g-w}\right)rect\left(\frac{x}{g-w}\right)\right]**\sum_m\sum_n \delta(x-ng)\delta(y-mg)\right\} \tag{3}$$

Where *g* is the metal line width, *w* is the line spacing, *Ng* refers to the sample diameter, $rect(x)rect(y)$ is a 2D rectangle function, $circ(r)$ is a circle function, ** is a 2D convolution, $\sum_m\sum_n \delta(x-ng)\delta(y-mg)$ is a 2D comb function.

The intensity point spread function (PSF) of an optical system is the modulus squared of the Fourier transform of its pupil function.[7, 8] Taking the whole sample as a circular aperture, and the normalized intensity PSF of the square mesh can be given by the following equation:

$$I_s(\xi,\eta) = r^4 \sum_m \sum_n \left[\sin c(rn) \sin c(rm)\right]^2 \left[\frac{J_1\left[\pi Ng\sqrt{(\xi - n/g)^2 + (\eta - m/g)^2}\right]}{2Ng\sqrt{(\xi - n/g)^2 + (\eta - m/g)^2}} \right]^2 \quad (4)$$

here r is the duty ratio of metal lines, and it is defined as $r = (g\text{-}w)/g$, $J_1(\rho)$ is the first-order Bessel function. According to Eq. (4), normalized diffraction intensity can be calculated for each-order diffraction points, as shown in Supplementary Fig. 8.

Supplementary Note 4. Diffraction Calculation based on image processing

In the region of Fresnel diffraction (or equivalently, in the far field), the observed field strength U(x, y) can be represented as[9]:

$$\begin{aligned} U(x,y) &= \frac{1}{j\lambda z}\exp(jkz)\exp[j\frac{k}{2z}(x^2+y^2)] \times \iint U(x_0,y_0)\exp[-j\frac{k}{2z}(xx_0+yy_0)]dx_0dy_0 \\ &= \frac{1}{j\lambda z}\exp(jkz)\exp[j\frac{k}{2z}(x^2+y^2)] \times \mathcal{F}\{U(x_0,y_0)\}\Big|_{f_x=\frac{x}{\lambda z}, f_y=\frac{y}{\lambda z}} \end{aligned} \quad (5)$$

which shows that the observed field strength is proportional to the Fourier transform of the aperture distribution. The diffraction intensity distribution of the observed field can further be evaluated as:

$$I(x,y) = \left(\frac{1}{\lambda z}\right)^2 \left|\mathcal{F}\{U(x_0,y_0)\}\right|^2 = \left(\frac{1}{\lambda z}\right)^2 \left|A_0\left(\frac{x}{\lambda z},\frac{y}{\lambda z}\right)\right|^2 \quad (6)$$

Here, A_0 is the frequency spectrum of complex amplitude distribution of the aperture field. Neglecting constant coefficients, the diffraction intensity distribution equals to the power spectrum of the aperture field distribution.

Supplementary Note 5. System SNR analysis of metallic mesh

In the imaging application, the flux contained in the central order (zeroth-order) represents the signal. The total flux reaching a given image point results from both the signal and background (noise). For a given point in the scene, the fraction of incident energy that is diffracted away from the central order into the background is obviously the total transmittance of the mesh (T_{opt}) minus the transmittance to the central order ($T_{(0,0)}$). If the scene is fairly uniform and extended, the unwanted background component will also be fairly uniform and extended across the image. Thus, the ratio of the system signal-to-background level is

$$SNR = \frac{\text{signal}}{\text{background}} = \frac{T_{(0,0)}}{T_{opt} - T_{(0,0)}} \tag{7}$$

because of the inclusion of a mesh in the optical system. For a square mesh, the transmittance to the central order is reduced by T_{opt}^2, the signal-to-background ratio is reduced to

$$SNR_{\text{square mesh}} = \frac{T_{(0,0)}}{T_{opt} - T_{(0,0)}} = \frac{T_{opt}}{1 - T_{opt}} \tag{8}$$

Supplementary Tables

Supplementary Table 1. Ingredients for the CE3

Ingredients	MMA	BA	AA	HPA	APS	SDS	OP-10	$NaHCO_3$	ammonia	water
Mass [g]	30	10	1.5	3	0.2	0.8	1.2	0.5	1	55

Supplementary Table 2. Monomer dosages for the CE solutions with different values of $R_{MM\text{-}B}$

CE	RMM-B	MMA [g]	BA [g]
-	1:1	20	20
CE2	2:1	26.7	13.3
CE3	3:1	30	10
CE7	7:1	35	5

Supplementary References:

1. Preston, C., *et al*. Silver nanowire transparent conducting paper-based electrode with high optical haze. *J. Mater. Chem. C* **2**, 1248-1254 (2014).
2. Rao, K. D. M., Gupta R., Kulkarni G. U. Fabrication of Large Area, High-Performance, Transparent Conducting Electrodes Using a Spontaneously Formed Crackle Network as Template. *Adv. Mater. Interfaces*, (2014).
3. Kiruthika, S., *et al.* Large area solution processed transparent conducting electrode based on highly interconnected Cu wire network. *J. Mater. Chem. C* **2,** 2089-2094 (2014).
4. Chern, C. S. Emulsion polymerization mechanisms and kinetics. *Prog. Polym. Sci.* **31,** 443-486 (2006).
5. Tirumkudulu, M. S., Russel W. B. Cracking in drying latex films. *Langmuir* **21,** 4938-4948 (2005).
6. Singh, K. B., Tirumkudulu M. S. Cracking in drying colloidal films. *Phys. Rev. Lett.* **98,** (2007).
7. Tan, J. B., Lu Z. G. Contiguous metallic rings: an inductive mesh with high transmissivity, strong electromagnetic shielding, and uniformly distributed stray light. *Opt. Express* **15,** 790-796 (2007).
8. Kohin, M., Wein S. J., Traylor J. D., Chase R. C., Chapmant J. E. Analysis and design of transparent conductive coatings and filters. *Opt. Eng.* **32,** 911-925 (1993).
9. Goodman, J. W. *Introduction to Fourier Optics*. 2nd ed. McGraw-Hill: San Francisco, 63-95 (1996).
